# Supplementary figures and images for: Association between serum lipids concentration and patients with age-related cataract in China: a cross-sectional, case–control study
Source: BMJ Open. 2018 Apr 5;8(4):e021496. doi: 10.1136/bmjopen-2018-021496 (PMC5892756; doi:10.1136/bmjopen-2018-021496)

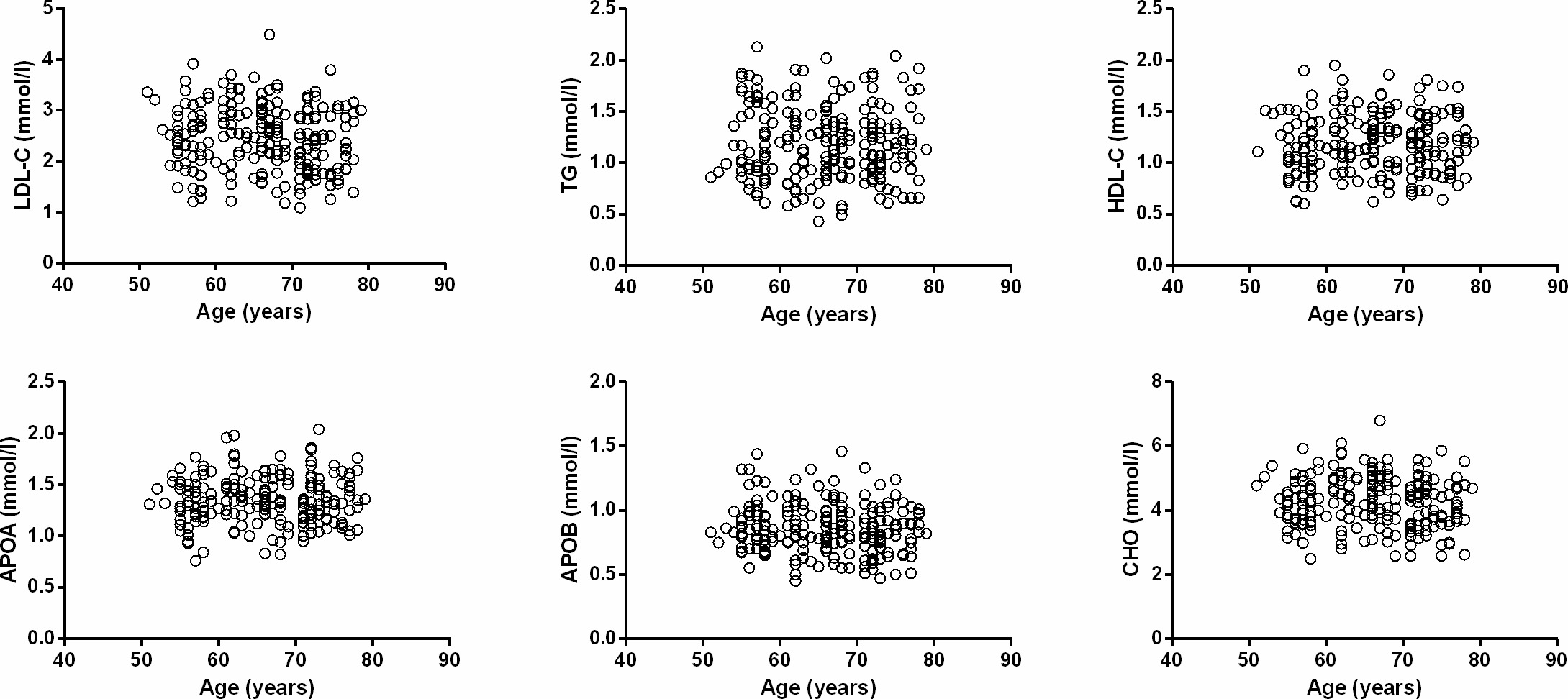

Supplement: Supplementary file 1 [file bmjopen-2018-021496supp001.jpg]
