# Supplementary material for: Association between serum lipids concentration and patients with age-related cataract in China: a cross-sectional, case–control study
Source: BMJ Open. 2018 Apr 5;8(4):e021496. doi: 10.1136/bmjopen-2018-021496 (PMC5892756; doi:10.1136/bmjopen-2018-021496)
Supplement: Author's manuscript [file bmjopen-2018-021496.draft_revisions.pdf]

BMJ Open

BMJ Open is committed to open peer review. As part of this commitment we make the peer review history of every article we publish publicly available.

When an article is published we post the peer reviewers' comments and the authors' responses online. We also post the versions of the paper that were used during peer review. These are the versions that the peer review comments apply to.

The versions of the paper that follow are the versions that were submitted during the peer review process. They are not the versions of record or the final published versions. They should not be cited or distributed as the published version of this manuscript.

BMJ Open is an open access journal and the full, final, typeset and author-corrected version of record of the manuscript is available on our site with no access controls, subscription charges or pay-per-view fees (http://bmjopen.bmj.com).

If you have any questions on BMJ Open's open peer review process please email info.bmjopen@bmj.com

BMJ Open

Association between serum lipids concentration and patients with age-related cataract: a cross-sectional, case-control study

Journal:	*BMJ Open*
Manuscript ID	bmjopen-2018-021496
Article Type:	Research
Date Submitted by the Author:	04-Jan-2018
Complete List of Authors:	Li, shengjie; Eye & ENT Hospital, Shanghai Medical College, Fudan University, Department of Clinical Laboratory<br>li, dan; Xi'an Jiaotong University School of Medicine, Key Laboratory of Environment and Genes Related to Diseases, Ministry of Education<br>zhang, yudong; Eye & ENT Hospital, Shanghai Medical College, Fudan University, Department of Clinical Laboratory<br>teng, jisen; Eye & ENT Hospital, Shanghai Medical College, Fudan University, Department of Clinical Laboratory<br>shao, mingxi; Eye & ENT Hospital, Shanghai Medical College, Fudan University, Department of Clinical Laboratory<br>cao, wenjun; Eye & ENT Hospital, Shanghai Medical College, Fudan University, Department of Clinical Laboratory
Keywords:	age-related cataract, serum lipid, case-control study, low-density lipoprotein cholesterol, triglyceride

SCHOLARONE™
Manuscripts

only

Association between serum lipids concentration and patients with age-related cataract: a cross-sectional, case-control study

Shengjie Li[1,3], Danhui Li[2], Yudong Zhang[1], Jisen Teng[1], Mingxi Shao[1], Wenjun Cao[1,3]

1. Department of Clinical Laboratory, Eye & ENT Hospital, Shanghai Medical College, Fudan University, 200031, Shanghai, China

2. Key Laboratory of Environment and Genes Related to Diseases, Ministry of Education, Medical School, Xi'an Jiaotong University, 710000, Xi'an, Shanxi, China

3. Department of Ophthalmology & Visual Science, Eye & ENT Hospital, Shanghai Medical College, Fudan University, 200031,Shanghai, China

Corresponding author: Wenjun Cao, E-mail:wgkjyk@aliyun.com

Mingxi Shao, E-mail:smingxi@sohu.com

Mingxi Shao and Wenjun Cao contributed equally to this paper.

Tel: 021-64377134

Address: No.83 Fenyang Road, Shanghai, China, 200031

Funding: This research project was supported by the Shanghai Municipal Commission of Health and Family Planning (20174Y0169).

Conflict of Interest: There were no conflicting relationships for any author.

Ethics approval: The ethics committee of the Eye and ENT Hospital, Shanghai Medical College approved this study.

Data sharing statement: No additional data are available.

Author's contribution: Shengjie Li, Wenjun Cao, and Mingxi Shao were responsible for the conception and design of the manuscript. Shengjie Li and Danhui Li analyzed and interpreted the data. Shengjie Li, Yudong Zhang, and Jisen Teng collected the data. Shengjie Li wrote the paper. All the authors critically reviewed the manuscript for scientific content and approved the final version.

For peer review only

Abstract

Objective: Obesity and a high-fat diet have been found to be associated with an increased risk of age-related cataract (ARC). Thus, this study aimed to investigate whether serum lipid levels are associated with the incidence of ARC.

Design: Cross-sectional, case-control study.

Setting: Eye & ENT Hospital of Fudan University, Shanghai, China.

Participants: A total of 219 ARC (male = 94, female = 125) subjects and 218 (male = 110, female = 118) normal control subjects were recruited in this study.

Outcome measures: A detailed eye and systematic examination was performed. Serum high-density lipoprotein cholesterol (HDL-C), low-density lipoprotein cholesterol (LDL-C), triglyceride (TG), and cholesterol (CHO) levels were measured by enzymatic colorimetry, and serum apolipoprotein A (APOA) and apoB (APOB) levels were measured by immunoturbidimetry. The subgroups were classified according to gender and types of disease (cortical, nuclear, and posterior subcapsular cataract). Logistic regression analyses were performed to identify the association between serum lipid levels and ARC.

Results: The serum LDL-C, TG, CHO, and APOA levels were significantly higher ($p < 0.05$) in the ARC group than in the control group. A similar result was observed when the serum lipid concentrations were compared between the ARC and control groups both in male and female subgroups. A higher proportion of individuals in the ARC group had higher LDL-C, TG, CHO, and APOA levels (fold = 3.45, 17.37, 3.27, and11.91, respectively; $p < 0.0001$ in all cases) than in the control group. Results of the logistic regression analyses revealed that high LDL-C (Odds ratios [ORs] = 1.897, 95% CI = 0.960 – 3.678), and TG (OR = 1.854, 95% CI = 1.232 – 2.791) were the independent risk factors for ARC.

Conclusion: The serum LDL-C and TG levels were significantly higher in patients with ARC, and both parameters were further demonstrated to be independent risk factors for ARC.

For peer review only

Strengths and limitations of this study

1. This is the first study focusing on the evaluation of the serum lipid concentration in ARC.

2. Serum LDL-cholesterol and triglycerides, but not HDL-cholesterol, are associated with an increased risk of age-related cataract.

3. The detailed demographic and clinical data of studied subjects.

4. The subjects involved in the study are from a racially homogeneous background (all Chinese).

5. We did not consider treatment with lipid-lowering medications and how it might affect the results.

Introduction

Age-related cataract (ARC), one of the leading causes of blindness and poor vision worldwide[1], remains a severe public health challenge worldwide, particularly in China[2]. There are three types of ARC based on the location of the opacity within the lens: cortical, nuclear, and posterior subcapsular (PSC)[3]. Surgery, which is the only available treatment for ARC, is a major medical cost given China's rapidly aging populations and large numbers of ARC patients. ARC is a complex diseases with multiple risk components, including high myopia[2], diabetes[4], hyperglycemia[5], obesity[6], blood pressure[4], lipids[7]. Among these risk factors, obesity, blood pressure, and lipids have been found to be consistently associated with blood lipid concentration.

Whether elevated blood lipid concentration is a risk factor for ARC is uncertain. Hiller et al.[7] reported that fasting hypertriglyceridemia (≥250 mg/dL) is associated with an increased risk of PSC cataract in men. In a population-based, cross-sectional study, Tang et al.[2] found that high low-density lipoprotein and low high-density lipoprotein were independent risk factors for ARC. However, Marks RG et al.[8] reported that both men and women with cataracts had significantly lower serum cholesterol (CHO) concentrations than subjects without cataracts. The relationship between blood lipid concentration and ARC risk is less consistent. Some have suggested that blood lipid concentration is an increased risk factor for ARC[5,9,10], and

For peer review only

others have shown the opposite effect[8,11] or no association[12]. Here, we conducted a large, cross-sectional, case-control study to detect and compare the blood lipid levels between ARC patients and normal subjects and to investigate the possible association between blood lipid levels and the risk of ARC.

Methods

Patients

This study was approved by the Ethics Committee of the Eye & ENT Hospital of Fudan University, Shanghai, and complied with the Declaration of Helsinki. Informed consent was obtained from all the subjects. Patients with ARC were consecutively recruited from the Department of Ophthalmology &Visual Science, Eye & ENT Hospital of Fudan University between June 2016 and June 2017. Both newly diagnosed and referral ARC patients were included. Normal controls were consecutively recruited from individuals who participated in annual health screenings during the study period.

Medical examinations included assessments of electrocardiograms, X-rays, liver function, renal function, infectious disease, blood pressure, heart rate, and body temperature. All subjects underwent medical examinations at the Eye and ENT Hospital of Fudan University. Alcohol consumption (>3 times per week for more than six months), smoking (>1 cigarette per day and more than six months [current or former]) (self-reported), and dietary habits (meat [less vegetables]/vegetables [less meat]) were also collected. Furthermore, each ARC patient underwent a standardized ophthalmic examination performed by cataract specialists. A visual acuity measurement was obtained for each patient based on the International Standard Visual Acuity Chart. The details of the standardized ophthalmic examination have been previously described[13].

Inclusion criteria

ARC subjects

A total of 320 cataract subjects were recruited, of whom 101 (metabolic cataract = 15, traumatic cataract = 8, congenital cataract = 17, secondary cataract = 34, cancer = 3, renal disease = 3, liver disease = 5, other eye diseases = 16) were excluded from the

For peer review only - http://bmjopen.bmj.com/site/about/guidelines.xhtml

study based on the inclusion criteria. If both eyes suffered from ARC, only one eye was randomly selected for the study. The final sample consisted of 219 ARC patients who met the following inclusion criteria as described previously[13]: age ≥ 45 years; ARC recorded using the Lens Opacities Classification System III (LOCS III); ARC defined as the presence of any type of ARC (cortical, nuclear, or PSC) or a history of previous cataract surgery (pseudophakic or aphakic eyes) in either eye; no history of secondary cataracts, congenital cataracts, or any other eye diseases; and no history of systemic diseases such as acute infectious diseases, kidney disease, autoimmune disease, and cancer.

Control subjects

A total of 250 subjects were recruited. Of these, 32 normal subjects (cataract = 5, cancer = 2, renal disease = 6, liver disease = 4, autoimmune disease = 2, macular degeneration = 3, cardiovascular disease = 4, other diseases = 6) were later excluded from the study based on the inclusion criteria. The final sample consisted of 218 control subjects who met the following previously described criteria[13]: age ≥ 45 years; no history of any types of cataracts, secondary cataracts, congenital cataracts, and any other eye diseases; and no history of systemic diseases such as acute infectious diseases, kidney disease, autoimmune disease, and cancer.

Data collection

Clinical and demographic information were obtained from the medical data platform of Eye-ENT Hospital by trained staff (Shengjie Li and Mingxi Shao) using standardized data collection and quality control procedures, which produced reliable data for analysis. Moreover, the study clerks conducted face-to-face questionnaire interviews to gather data such as life habits, tobacco and alcohol intake, height, and body weight.

For serum lipid measurements, blood samples were obtained in the morning after the subjects had fasted for 8 hours via standard venipuncture of the veins in the antecubital fossae. Blood coagulation was prevented using EDTA, and the obtained samples were centrifuged within 60 min (3000 rpm, 10 minutes, 20°C). Serum high-density lipoprotein cholesterol (HDL-C), low-density lipoprotein cholesterol

For peer review only

(LDL-C), TG, and cholesterol (CHO) levels were measured by enzymatic colorimetry using a commercially available kit (Roche Diagnostics GmbH, Mannheim, Germany). Serum apolipoprotein A (APOA) and B (APOB) levels were measured by immunoturbidimetry using a commercially available kit (Roche Diagnostics, GmbH, Mannheim, Germany). Internal controls were analyzed daily over a 2-year period, with typical monthly coefficient of variation of 2–5% and no significant changes in the values.

Statistical analyses

The results of this study are presented as mean ± standard deviation. Independent student's *t* test and chi-square test were used to compare the subject characteristics between the ARC and control groups. One-way analysis of variance was used to compare the serum lipid levels among the three groups (cortical, nuclear, and PSC). Based on the normal reference range value of LDL-C (LDL-C ≥ 3.36 mmol/L, LDL-C < 3.36 mmol/L), TG (TG⩾2.26mmol/l, TG < 2.26 mmol/L), CHO (CHO⩾ 5.20mmol/l, CHO<5.20mmol/l), and APOA (APOA ≥ 1.9 mmol/L, APOA < 1.9 mmol/L), the ARC and control subjects were divided into lower and higher groups, respectively. The prevalence of ARC in China is higher in females than in males (37.2% in men and 39.0% in women)[2]. Moreover, in our study, there were more female ARC subjects than male ARC subjects (125 vs. 94). Therefore, the subjects were categorized into gender subgroups. A two-sided $P < 0.05$ was considered statistically significant. Logistic regression analyses were performed to identify the association between serum lipid levels and ARC (control group = 0, ARC = 1; male = 0, female = 1). Odds ratios (ORs) with 95% confidence intervals (95%CI) were estimated by logistic regression analyses. All analyses were performed using SPSS 13.0 software (SPSS Inc., Chicago, IL, USA).

Results

A total of 219 ARC (male = 94, female = 125) subjects and 218 (male = 110, female = 118) normal control subjects were recruited in this study. There was no significant difference in the mean age, gender, smoking, and drinking between the ARC and control groups ($p > 0.005$). However, there was a significant difference in the body

For peer review only - http://bmjopen.bmj.com/site/about/guidelines.xhtml

mass index (BMI), systolic blood pressure, diastolic blood pressure, and diet between the ARC and control groups ($p < 0.05$). Table 1 presents a summary of the demographics of the ARC and control groups.

Comparison of serum HDL-C, LDL-C, TG, CHO, APOA, and APOB levels between the ARC and control subjects

Table 2 and Figure 1 show the HDL-C, LDL-C, TG, CHO, APOA, and APOB levels in the ARC group. The level of serum LDL-C, TG, CHO, and APOA was significantly higher ($p < 0.05$) in the ARC group than in the control group.

Moreover, the ARC and control subjects were categorized into female and male subgroups. In both the female and male subgroups, the TG level was significantly higher ($p < 0.05$) in the ARC group than in the control group. In the male subgroup, the APOA level was significantly higher ($p < 0.05$) in the ARC group than in the control group. In the female subgroup, the level of LDL-C was significantly higher ($p < 0.05$) in the ARC group than in the control group.

Serum LDL-C, TG, CHO, and APOA levels in the ARC and control groups

The ARC and control subjects were each divided into a lower group and a higher group according to whether their serum LDL-C, TG, CHO, and APOA values were below or above the normal reference range, respectively (Table 3 and Figure 2). Compared to the control group, more subjects from the ARC group were in the higher group (3.45, 17.37, 3.27, and11.91 fold, respectively, $p < 0.0001$).

Comparison of serum lipid levels in subjects with different types of ARC

Of the 219 ARC patients, 85 had nuclear cataracts, 108 had cortical cataracts, and 26 had PSC. Table 4 presents a comparison of the serum lipid levels and the demographic parameters in the ARC subjects. There were no statistically significant differences in the demographic and serum lipid levels among the nuclear, cortical, and PSC cataract groups ($p > 0.05$).

Logistic regression analysis of the association between serum lipid levels and ARC

Logistic regression analyses were performed to identify the association between serum HDL-C, LDL-C, TG, CHO, APOA, and APOB levels and ARC in comparison

For peer review only

to subjects without ARC (Table 5). Logistic regression analyses revealed that being female (OR = 1.719, 95% CI = 1.074–2.752) and BMI (OR = 1.297, 95% CI = 1.194–1.408), systolic blood pressure (OR = 1.029, 95% CI = 1.008–1.051), LDL-C (OR = 1.897, 95% CI = 0.960–3.678), and TG levels (OR = 1.854, 95% CI = 1.232–2.791) were associated with ARC after adjusting for age and other demographic parameters.

Discussion

In this case-control study of the Chinese Han population, we found the following: First, the serum LDL-C, TG, CHO, and APOA levels was significantly higher ($p < 0.05$) in the ARC group than in the control group. Second, the proportion of individuals with higher LDL-C, TG, CHO, and APOA levels was higher in the ARC group (3.45, 17.37, 3.27, and 11.91-fold, $p < 0.0001$) relative to the control group. Finally, high serum LDL-C (OR = 1.897, 95% CI = 0.960–3.678), and TG levels (OR = 1.854, 95% CI = 1.232–2.791) were associated with ARC after adjusting for age, gender, and other demographic parameters. The results of this case-control study indicate that higher serum LDL-C and TG levels are associated with a significantly increased risk of ARC.

In a study on the association of metabolic syndrome and ARC, reduced HDL-C and elevated TG levels were found to be significantly associated with cataract in women[14]. In a population-based study (n = 4,926) in Beaver Dam, Klein et al.[15] reported that higher serum HDL-C levels were associated with decreased risk of cataract. Moreover, an extremely strong association ($p < 0.0001$) between HDL-C levels and the development of lens opacities was also reported in the South African population[16]. Furthermore, an elevated level of serum TG was significantly related to cataract in some studies[7,17]. In the present study, we also found that serum TG concentration was higher in the ARC group than in the normal group and that TG concentration was a risk factor of ARC, but the HDL-C level did not significantly differ between groups. Sabanayagam et al.[4] also found no significant associations between serum HDL-C and cataract. The mechanism linking hyperlipidemia and cataract is not clear. Thus far, no studies have clarified how changes in blood lipid affect the onset and/or development

For peer review only - http://bmjopen.bmj.com/site/about/guidelines.xhtml

of ARC. Animal studies have shown that hyperlipidemia and low HDL-C are associated with an earlier onset and an elevated incidence of diabetic cataracts[18], and inflammation and oxidative stress resulting from low HDL-C levels could induce cataract formation[19]. Moreover, it has been suggested that hyperlipidemia is related to ARC through its associated complications such as cardiovascular disease[9,15].

In the female and male ARC subgroups, the TG level was significantly higher ($p < 0.05$) in the ARC group than in the control group. However, the APOA level was significantly higher in the ARC group than in the control group in the male subgroup but not in the female subgroup. In the female subgroup, the LDL-C level was significantly higher in the ARC group than in the control group but not in the male subgroup. Tavani et al.[20] reported that hyperlipidemia (OR, 1.8; 95% CI, 1.2–2.7) was associated with an increased risk of cataract in women from northern Italy. In addition, elevated TG was also significantly associated with cataract in women but not in men[14]. A consistent gender difference in the association between blood lipid concentration and cataract was observed. Differences of hormonal[21] and life-style related characteristics[22] between men and women may explain this gender difference.

In the subgroup analysis for ARC subtype, no statistically significant difference in the demographic and serum lipid levels was found among the nuclear, cortical, and PSC cataract groups ($p > 0.05$). Hiller et al.[7] found that HDL-C levels were associated with PSC cataract but not in cortical and nuclear cataract. Moreover, Park et al.[14] reported that reduced HDL-C was only significantly associated with nuclear cataract. The results of these existing studies are controversial, and the potential pathogenesis was unclear.

Our study also found that female gender (OR = 1.719, 95% CI = 1.074–2.752), high BMI (OR = 1.297, 95% CI = 1.194–1.408), and high systolic blood pressure (OR = 1.029, 95% CI = 1.008–1.051), which were closely associated with serum lipid concentration, were associated with an increased risk of ARC after adjusting for age and other demographic parameters. In the Blue Mountains Eye Study, obesity (BMI ≥ 30 kg/m^2) and hypertension were significantly associated with an increased incidence of cataract[23]. Moreover, Jacques et al.[6] also reported that women with a

BMI of ≥30 had a higher prevalence of PSC opacities than women with a BMI of <25 (OR: 2.5).

We acknowledge that our study has some limitations. First, as this is a cross-sectional, case-control study, our ability to explore the exact mechanisms underlying the associations between serum lipid concentration and ARC remained limited. This in turn limited our ability to make exact causal inferences between serum lipid concentration and ARC. Second, the subjects involved in the study are from a racially homogeneous background (all Chinese), which may limit the generalizability of the results. Third, we did not consider treatment with lipid-lowering medications and how it might affect the results. Therefore, a multicenter study with a larger sample size should be conducted in the future.

In conclusion, the findings from this case-control study suggest that the serum LDL-C, TG, CHO, and APOA concentrations were significantly higher in the ARC patients than in the controls. The serum LDL-C and TG concentration were significantly associated with ARC. In addition, no significant difference in the serum lipid levels was found between the nuclear, cortical, and PSC cataract groups. These findings indicate a need for health promotional activities aimed at controlling blood lipid concentration among high-risk populations.

References

1. Bourne RRA, Stevens GA, White RA, et al. Causes of vision loss worldwide, 1990-2010: a systematic analysis. *Lancet Glob Health*. 2013;1(6):e339-349. doi:10.1016/S2214-109X(13)70113-X.

2. Tang Y, Wang X, Wang J, et al. Prevalence of Age-Related Cataract and Cataract Surgery in a Chinese Adult Population: The Taizhou Eye Study. *Invest Ophthalmol Vis Sci*. 2016;57(3):1193-1200. doi:10.1167/iovs.15-18380.

3. Klein BE, Klein R, Linton KL. Prevalence of age-related lens opacities in a population. The Beaver Dam Eye Study. *Ophthalmology*. 1992;99(4):546-552.

4. Sabanayagam C, Wang JJ, Mitchell P, et al. Metabolic syndrome components and age-related cataract: the Singapore Malay eye study. *Invest Ophthalmol Vis Sci*. 2011;52(5):2397-2404. doi:10.1167/iovs.10-6373.

5. Hennis A, Wu S-Y, Nemesure B, Leske MC, Barbados Eye Studies Group. Risk factors for incident cortical and posterior subcapsular lens opacities in the

For peer review only

Barbados Eye Studies. *Arch Ophthalmol Chic Ill 1960*. 2004;122(4):525-530. doi:10.1001/archopht.122.4.525.

6. Jacques PF, Moeller SM, Hankinson SE, et al. Weight status, abdominal adiposity, diabetes, and early age-related lens opacities. *Am J Clin Nutr*. 2003;78(3):400-405.

7. Hiller R, Sperduto RD, Reed GF, D'Agostino RB, Wilson PWF. Serum lipids and age-related lens opacities: a longitudinal investigation: the Framingham Studies. *Ophthalmology*. 2003;110(3):578-583. doi:10.1016/S0161-6420(02)01762-1.

8. Marks RG, Hale WE, Perkins LL, May FE, Stewart RB. Cataracts in Dunedin Program participants: an evaluation of risk factors. *J Cataract Refract Surg*. 1988;14(1):58-63.

9. Jahn CE, Janke M, Winowski H, von Bergmann K, Leiss O, Hockwin O. Identification of metabolic risk factors for posterior subcapsular cataract. *Ophthalmic Res*. 1986;18(2):112-116.

10. Heydari B, Kazemi T, Zarban A, Ghahramani S. Correlation of cataract with serum lipids, glucose and antioxidant activities: a case-control study. *West Indian Med J*. 2012;61(3):230-234.

11. Clayton RM, Cuthbert J, Duffy J, et al. Some risk factors associated with cataract in S.E. Scotland: a pilot study. *Trans Ophthalmol Soc U K*. 1982;102 Pt 3:331-336.

12. Klein BE, Klein R, Lee KE. Diabetes, cardiovascular disease, selected cardiovascular disease risk factors, and the 5-year incidence of age-related cataract and progression of lens opacities: the Beaver Dam Eye Study. *Am J Ophthalmol*. 1998;126(6):782-790.

13. Shao M, Li D, Teng J, Zhang Y, Li S, Cao W. Association Between Serum Complement C3 Levels and Age-Related Cataract. *Invest Ophthalmol Vis Sci*. 2017;58(11):4934-4939. doi:10.1167/iovs.17-22522.

14. Park Y-H, Shin JA, Han K, Yim HW, Lee W-C, Park Y-M. Gender difference in the association of metabolic syndrome and its components with age-related cataract: the Korea National Health and Nutrition Examination Survey 2008-2010. *PloS One*. 2014;9(1):e85068. doi:10.1371/journal.pone.0085068.

15. Klein BE, Klein R, Lee KE. Cardiovascular disease, selected cardiovascular disease risk factors, and age-related cataracts: the Beaver Dam Eye Study. *Am J Ophthalmol*. 1997;123(3):338-346.

16. Meyer D, Parkin D, Maritz FJ, Liebenberg PH. Abnormal serum lipoprotein

For peer review only

levels as a risk factor for the development of human lenticular opacities. *Cardiovasc J South Afr Off J South Afr Card Soc South Afr Soc Card Pract*. 2003;14(2):60-64.

17. Paunksnis A, Bojarskiene F, Cimbalas A, Cerniauskiene LR, Luksiene DI, Tamosiunas A. Relation between cataract and metabolic syndrome and its components. *Eur J Ophthalmol*. 2007;17(4):605-614.

18. Tsutsumi K, Inoue Y, Yoshida C. Acceleration of development of diabetic cataract by hyperlipidemia and low high-density lipoprotein in rats. *Biol Pharm Bull*. 1999;22(1):37-41.

19. von Eckardstein A, Hersberger M, Rohrer L. Current understanding of the metabolism and biological actions of HDL. *Curr Opin Clin Nutr Metab Care*. 2005;8(2):147-152.

20. Tavani A, Negri E, La Vecchia C. Selected diseases and risk of cataract in women. A case-control study from northern Italy. *Ann Epidemiol*. 1995;5(3):234-238.

21. Baños G, Guarner V, Pérez-Torres I. Sex steroid hormones, cardiovascular diseases and the metabolic syndrome. *Cardiovasc Hematol Agents Med Chem*. 2011;9(3):137-146.

22. Lee DH, Kim YM, Jekal Y, et al. Low levels of physical activity are associated with increased metabolic syndrome risk factors in korean adults. *Diabetes Metab J*. 2013;37(2):132-139. doi:10.4093/dmj.2013.37.2.132.

23. al YC et. Cardiovascular disease, vascular risk factors and the incidence of cataract and cataract surgery: the Blue Mountains Eye Study. - PubMed - NCBI. https://www.ncbi.nlm.nih.gov/pubmed/14628965. Accessed November 18, 2017.

For peer review only

Figure Legends

Figure 1. Comparison of serum lipid levels in age-related cataract (ARC) and the control group. Top of the box plot represents the mean and the bar of each box represents the standard deviation. LDL-C: low density lipoprotein cholesterol, TG: triglyceride, APOA: apoA apolipoprotein, CHO: cholesterol.

Figure 2. The number of subjects in the age-related cataract (ARC) and control groups, according to normal reference range value of low density lipoprotein cholesterol (LDL-C), triglyceride (TG), cholesterol (CHO), and apoA apolipoprotein (APOA) levels.

For peer review only

Tables

Table 1. Demographics of subjects between ARC and control group

	ARC group (n=219)	Control group (n=218)	t value	P value
Age (year)	66.99±8.83	66.00±7.10	1.281	0.201
Gender (Male/Female)	94/125	110/118	1.276	0.259
BMI (kg/m^2)	23.68±3.16	21.55±2.74	7.476	<0.001
SBP (mmHg)	132.45±15.50	129.92±5.55	2.230	0.027
SDP (mmHg)	74.21±10.65	76.58±5.37	2.896	0.004
IOP (mmHg)	13.84±3.59	-	-	-
Vision	0.23±0.15	-	-	-
Smoking (yes/no)	15/204	18/200	0.310	0.578
Drinking (yes/no)	33/186	23/195	1.996	0.158
Diet (meat/vegetarian)	159/60	98/120	34.476	<0.001

ARC: age-related cataract, BMI: body mass index, SBP: systolic blood pressure, SDP: diastolic blood pressure, IOP: intraocular pressure. Data are expressed as mean±standard deviation (SD). Independent-Samples T Test and chi-square test was used.

Table 2. Comparison of serum lipid levels between ARC and control group, stratified according to gender

	ARC group	Control group	t value	P value
LDL-C (mmol/l)	2.65±0.93	2.46±0.64	2.461	0.014
Male	2.45±0.85	2.43±0.60	0.184	0.854
Female	2.82±0.97	2.49±0.67	2.894	0.004
TG (mmol/l)	1.56±1.25	1.18±0.35	4.238	<0.001
Male	1.54±1.42	1.15±0.36	2.609	0.010
Female	1.57±1.10	1.22±0.35	3.364	0.001
HDL-C (mmol/l)	1.14±0.43	1.19±0.28	1.201	0.231
Male	1.10±0.48	1.10±0.28	0.064	0.949
Female	1.18±0.39	1.28±0.25	2.158	0.032
APOA (mmol/l)	1.34±0.23	1.28±0.36	2.270	0.024
Male	1.30±0.23	1.22±0.36	1.983	0.048
Female	1.39±0.22	1.33±0.35	1.649	0.101
APOB (mmol/l)	0.85±0.26	0.86±0.19	0.772	0.441
Male	0.81±0.25	0.83±0.18	0.757	0.450
Female	0.87±0.26	0.89±0.19	0.590	0.556
CHO (mmol/l)	4.47±1.78	4.26±0.79	2.146	0.032
Male	4.21±1.10	4.03±0.69	1.438	0.152
Female	4.67±1.20	4.49±0.81	1.275	0.204

ARC: age-related cataract, LDL-C: low density lipoprotein cholesterol, HDL-C: high density lipoprotein cholesterol, TG: triglyceride, APOA: apoA apolipoprotein, APOB: apoB apolipoprotein, CHO: cholesterol. Data are expressed as mean±standard deviation (SD). Independent-Samples T Test was used.

For peer review only

Table 3. The number of subjects in ARC and control group, according to LDL-C, TG, CHO, and APOA levels, respectively

	ARC group	Control group	Fold (t value)	P value
LDL-C (mmol/l)				
<3.36	167	203		
⩾3.36	52 (52/219=23.74%)	15 (15/218=6.88%)	3.45 (17.818)	<0.001
TG (mmol/l)				
<2.26	184	216		
⩾2.26	35 (35/219=15.98%)	2 (2/218=0.92%)	17.37 (27.136)	<0.001
CHO (mmol/l)				
<5.20	153	198		
⩾5.20	66 (66/219=30.14%)	20 (20/218=9.17%)	3.27 (20.549)	<0.001
APOA (mmol/l)				
<1.9	207	217		
⩾1.9	12 (12/219=5.48%)	1 (1/218=0.46%)	11.91 (8.996)	<0.001

ARC: age-related cataract, LDL-C: low density lipoprotein cholesterol, TG: triglyceride, APOA: apoA apolipoprotein, CHO: cholesterol. Chi-square test was used.

Table 4. Comparison of serum lipid levels in subjects with different types of ARC

	Nuclear cataract	Cortical cataract	PSC	t value	P value
Age (year)	67.54±8.83	66.59±8.55	66.87±10.39	0.282	0.754
Gender (Male/Female)	33/52	49/59	12/14	0.985	0.619
BMI (kg/m^2)	23.62±3.24	23.65±3.14	24.13±3.04	0.219	0.804
SBP (mmHg)	134.28±16.65	131.15±14.38	131.63±16.61	1.006	0.367
SDP (mmHg)	74.22±8.28	73.65±10.67	77.94±18.93	1.132	0.324
LDL-C (mmol/l)	2.67±0.97	2.60±0.87	2.81±1.08	0.491	0.612
TG (mmol/l)	1.46±1.28	1.68±1.32	1.38±0.64	1.022	0.362
HDL-C (mmol/l)	1.18±0.39	1.11±0.45	1.18±0.46	0.784	0.458
APOA (mmol/l)	1.29±0.34	1.27±0.38	1.29±0.35	0.107	0.899
APOB (mmol/l)	0.85±0.26	0.83±0.25	0.87±0.31	0.341	0.712
CHO (mmol/l)	4.45±1.22	4.47±1.11	4.51±1.38	0.020	0.981

ARC: age-related cataract, PSC: posterior subcapsular, BMI: body mass index, SBP: systolic blood pressure, SDP: diastolic blood pressure, LDL-C: low density lipoprotein cholesterol, HDL-C: high density lipoprotein cholesterol, TG: triglyceride, APOA: apoA apolipoprotein, APOB: apoB apolipoprotein, CHO: cholesterol. Data are expressed as mean±standard deviation (SD). Independent-Samples T Test and chi-square test was used.

For peer review only

Table 5. Logistic regression analysis of the association between serum lipid levels and ARC

	B	OR	P value	95%CI
Female	0.542	1.719	0.024	1.074-2.752
BMI (kg/m^2)	0.260	1.297	<0.001	1.194-1.408
SBP (mmHg)	0.029	1.029	0.006	1.008-1.051
LDL-C (mmol/l)	0.631	1.897	0.046	0.960-3.678
TG (mmol/l)	0.617	1.854	0.003	1.232-2.791

ARC: age-related cataract, BMI: body mass index, SBP: systolic blood pressure, LDL-C: low density lipoprotein cholesterol, TG: triglyceride, 95%CI: 95%confidence interval. Logistic regression analyses were performed to identify the association between serum lipid levels and ARC (control group=0, ARC=1; male=0, female=1) after adjusting for age, smoking, drinking, diastolic blood pressure, diet, high density lipoprotein cholesterol, apoA apolipoprotein, apoB apolipoprotein, and cholesterol.

For peer review only

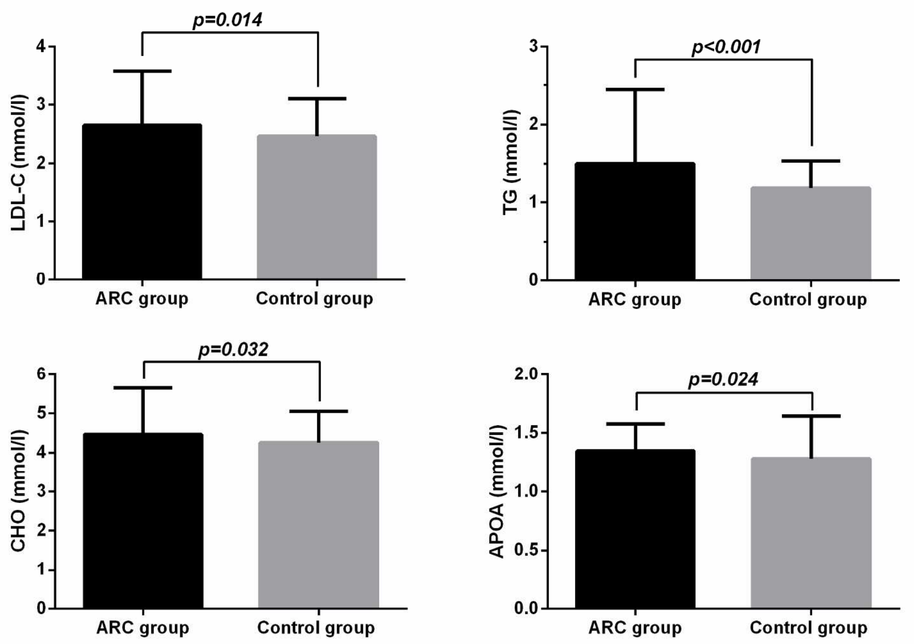

Figure 1

195x136mm (300 x 300 DPI)

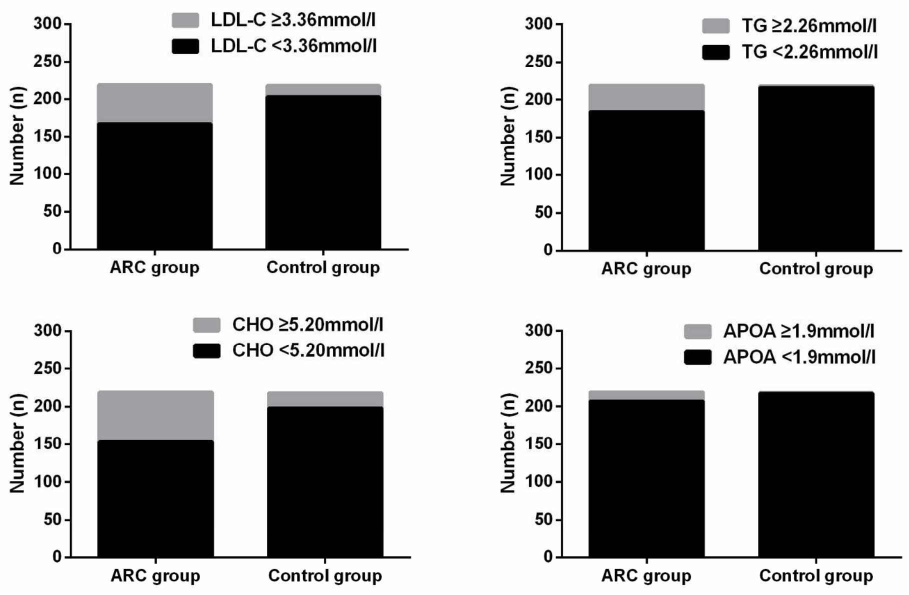

Figure 2

196x127mm (300 x 300 DPI)

For peer review only - http://bmjopen.bmj.com/site/about/guidelines.xhtml

CONSORT 2010 checklist of information to include when reporting a randomised trial*

Section/Topic	Item No	Checklist item	Reported on page No
**Title and abstract**			
	1a	Identification as a randomised trial in the title	1
	1b	Structured summary of trial design, methods, results, and conclusions (for specific guidance see CONSORT for abstracts)	1
**Introduction**			
Background and objectives	2a	Scientific background and explanation of rationale	2
	2b	Specific objectives or hypotheses	2-3
**Methods**			
Trial design	3a	Description of trial design (such as parallel, factorial) including allocation ratio	4
	3b	Important changes to methods after trial commencement (such as eligibility criteria), with reasons	4
Participants	4a	Eligibility criteria for participants	4-5
	4b	Settings and locations where the data were collected	4
Interventions	5	The interventions for each group with sufficient details to allow replication, including how and when they were actually administered	5
Outcomes	6a	Completely defined pre-specified primary and secondary outcome measures, including how and when they were assessed	5-6
	6b	Any changes to trial outcomes after the trial commenced, with reasons	4-6
Sample size	7a	How sample size was determined	4-5
	7b	When applicable, explanation of any interim analyses and stopping guidelines	4-5
Randomisation:			
Sequence generation	8a	Method used to generate the random allocation sequence	
	8b	Type of randomisation; details of any restriction (such as blocking and block size)	
Allocation concealment mechanism	9	Mechanism used to implement the random allocation sequence (such as sequentially numbered containers), describing any steps taken to conceal the sequence until interventions were assigned	
Implementation	10	Who generated the random allocation sequence, who enrolled participants, and who assigned participants to interventions	
Blinding	11a	If done, who was blinded after assignment to interventions (for example, participants, care providers, those	

For peer review only - http://bmjopen.bmj.com/site/about/guidelines.xhtml

		assessing outcomes) and how	
	11b	If relevant, description of the similarity of interventions	6
Statistical methods	12a	Statistical methods used to compare groups for primary and secondary outcomes	6
	12b	Methods for additional analyses, such as subgroup analyses and adjusted analyses	6
**Results**			
Participant flow (a diagram is strongly recommended)	13a	For each group, the numbers of participants who were randomly assigned, received intended treatment, and were analysed for the primary outcome	
	13b	For each group, losses and exclusions after randomisation, together with reasons	
Recruitment	14a	Dates defining the periods of recruitment and follow-up	
	14b	Why the trial ended or was stopped	
Baseline data	15	A table showing baseline demographic and clinical characteristics for each group	6,14
Numbers analysed	16	For each group, number of participants (denominator) included in each analysis and whether the analysis was by original assigned groups	6
Outcomes and estimation	17a	For each primary and secondary outcome, results for each group, and the estimated effect size and its precision (such as 95% confidence interval)	7
	17b	For binary outcomes, presentation of both absolute and relative effect sizes is recommended	7
Ancillary analyses	18	Results of any other analyses performed, including subgroup analyses and adjusted analyses, distinguishing pre-specified from exploratory	7
Harms	19	All important harms or unintended effects in each group (for specific guidance see CONSORT for harms)	
**Discussion**			
Limitations	20	Trial limitations, addressing sources of potential bias, imprecision, and, if relevant, multiplicity of analyses	10
Generalisability	21	Generalisability (external validity, applicability) of the trial findings	8-10
Interpretation	22	Interpretation consistent with results, balancing benefits and harms, and considering other relevant evidence	8-10
**Other information**			
Registration	23	Registration number and name of trial registry	
Protocol	24	Where the full trial protocol can be accessed, if available	
Funding	25	Sources of funding and other support (such as supply of drugs), role of funders	1

*We strongly recommend reading this statement in conjunction with the CONSORT 2010 Explanation and Elaboration for important clarifications on all the items. If relevant, we also recommend reading CONSORT extensions for cluster randomised trials, non-inferiority and equivalence trials, non-pharmacological treatments, herbal interventions, and pragmatic trials. Additional extensions are forthcoming: for those and for up to date references relevant to this checklist, see www.consort-statement.org.

For peer review only - http://bmjopen.bmj.com/site/about/guidelines.xhtml

BMJ Open

Association between serum lipids concentration and patients with age-related cataract in China: a cross-sectional, case-control study

Journal:	*BMJ Open*
Manuscript ID	bmjopen-2018-021496.R1
Article Type:	Research
Date Submitted by the Author:	21-Feb-2018
Complete List of Authors:	Li, shengjie; Eye & ENT Hospital, Shanghai Medical College, Fudan University, Department of Clinical Laboratory<br>li, dan; Xi'an Jiaotong University School of Medicine, Key Laboratory of Environment and Genes Related to Diseases, Ministry of Education<br>zhang, yudong; Eye & ENT Hospital, Shanghai Medical College, Fudan University, Department of Clinical Laboratory<br>teng, jisen; Eye & ENT Hospital, Shanghai Medical College, Fudan University, Department of Clinical Laboratory<br>shao, mingxi; Eye & ENT Hospital, Shanghai Medical College, Fudan University, Department of Clinical Laboratory<br>cao, wenjun; Eye & ENT Hospital, Shanghai Medical College, Fudan University, Department of Clinical Laboratory
**Primary Subject Heading**:	Ophthalmology
Secondary Subject Heading:	Ophthalmology
Keywords:	age-related cataract, serum lipid, case-control study, low-density lipoprotein cholesterol, triglyceride

SCHOLARONE™
Manuscripts

Association between serum lipids concentration and patients with age-related cataract in China: a cross-sectional, case-control study

Shengjie Li[1,3], Danhui Li[2], Yudong Zhang[1], Jisen Teng[1], Mingxi Shao[1], Wenjun Cao[1,3]

1. Department of Clinical Laboratory, Eye & ENT Hospital, Shanghai Medical College, Fudan University, 200031, Shanghai, China

2. Key Laboratory of Environment and Genes Related to Diseases, Ministry of Education, Medical School, Xi'an Jiaotong University, 710000, Xi'an, Shanxi, China

3. Department of Ophthalmology & Visual Science, Eye & ENT Hospital, Shanghai Medical College, Fudan University, 200031,Shanghai, China

Corresponding author: Wenjun Cao, E-mail:wgkjyk@aliyun.com

Mingxi Shao, E-mail:smingxi@sohu.com

Mingxi Shao and Wenjun Cao contributed equally to this paper.

Tel: 021-64377134

Address: No.83 Fenyang Road, Shanghai, China, 200031

Funding: This research project was supported by the Shanghai Municipal Commission of Health and Family Planning (20174Y0169).

Conflict of Interest: There were no conflicting relationships for any author.

Ethics approval: The ethics committee of the Eye and ENT Hospital, Shanghai Medical College approved this study (EENT2015011).

Data sharing statement: No additional data are available.

Author's contribution: Shengjie Li, Wenjun Cao, and Mingxi Shao were responsible for the conception and design of the manuscript. Shengjie Li and Danhui Li analyzed and interpreted the data. Shengjie Li, Yudong Zhang, and Jisen Teng collected the data. Shengjie Li wrote the paper. All the authors critically reviewed the manuscript for scientific content and approved the final version. The authors thank all the participants and the staff (the doctors, nurses, and administrative staff) at Eye-ENT Hospital, Shanghai Medical College, Fudan University for their contributions to this research.

For peer review only - http://bmjopen.bmj.com/site/about/guidelines.xhtml

Abstract

Objective: Obesity and a high-fat diet have been found to be associated with an increased risk of age-related cataract (ARC). Thus, this study aimed to investigate whether serum lipid levels are associated with the incidence of ARC.

Design: Cross-sectional, case-control study.

Setting: Eye & ENT Hospital of Fudan University, Shanghai, China.

Participants: A total of 219 ARC (male = 94, female = 125) subjects and 218 (male = 110, female = 118) normal control subjects were recruited in this study.

Outcome measures: A detailed eye and systematic examination was performed. Serum high-density lipoprotein cholesterol (HDL-C), low-density lipoprotein cholesterol (LDL-C), triglyceride (TG), and cholesterol (CHO) levels were measured by enzymatic colorimetry, and serum apolipoprotein A (APOA) and apoB (APOB) levels were measured by immunoturbidimetry. The subgroups were classified according to gender and types of disease (cortical, nuclear, and posterior subcapsular cataract). Logistic regression analyses were performed to identify the association between serum lipid levels and ARC.

Results: The serum LDL-C, TG, CHO, and APOA levels were significantly higher ($p < 0.05$) in the ARC group than in the control group. A similar result was observed when the serum lipid concentrations were compared between the ARC and control groups both in male and female subgroups. A higher proportion of individuals in the ARC group had higher LDL-C, TG, CHO, and APOA levels (fold = 3.45, 17.37, 3.27, and11.91, respectively; $p < 0.0001$ in all cases) than in the control group. Results of the logistic regression analyses revealed that high LDL-C (Odds ratios [ORs] = 1.897, 95% CI = 0.960 – 3.678), and TG (OR = 1.854, 95% CI = 1.232 – 2.791) were the independent risk factors for ARC.

Conclusion: The serum LDL-C and TG levels were demonstrated to be independent risk factors for ARC.

For peer review only - http://bmjopen.bmj.com/site/about/guidelines.xhtml

Strengths and limitations of this study

1. This study provides evidence for a better understanding on the association between serum lipids concentration and patients with age-related cataract.

2. The detailed demographic and clinical data of studied subjects.

3. The subjects involved in the study are from a racially homogeneous background (all Chinese).

4. We did not consider treatment with lipid-lowering medications and how it might affect the results.

Introduction

Age-related cataract (ARC), one of the leading causes of blindness and poor vision worldwide[1], remains a severe public health challenge worldwide, particularly in China[2]. There are three types of ARC based on the location of the opacity within the lens: cortical, nuclear, and posterior subcapsular (PSC)[3]. Surgery, which is the only available treatment for ARC, is a major medical cost given China's rapidly aging populations and large numbers of ARC patients. ARC is a complex diseases with multiple risk components, including high myopia[2], diabetes[4], hyperglycemia[5], obesity[6], blood pressure[4], lipids[7]. Among these risk factors, obesity, blood pressure, and lipids have been found to be consistently associated with blood lipid concentration.

Whether elevated blood lipid concentration is a risk factor for ARC is uncertain. Hiller et al.[7] reported that fasting hypertriglyceridemia (≥250 mg/dL) is associated with an increased risk of PSC cataract in men. In a population-based, cross-sectional study, Tang et al.[2] found that high low-density lipoprotein and low high-density lipoprotein were independent risk factors for ARC. However, Marks RG et al.[8] reported that both men and women with cataracts had significantly lower serum cholesterol (CHO) concentrations than subjects without cataracts. The relationship between blood lipid concentration and ARC risk is less consistent. Some have suggested that blood lipid concentration is an increased risk factor for ARC[5,9,10], and others have shown the opposite effect[8,11] or no association[12]. Here, we conducted a large, cross-sectional, case-control study to detect and compare the blood lipid levels

For peer review only - http://bmjopen.bmj.com/site/about/guidelines.xhtml

For peer review only

between ARC patients and normal subjects and to investigate the possible association between blood lipid levels and the risk of ARC.

Methods

Patient and Public Involvement statement

This study was approved by the Ethics Committee of the Eye & ENT Hospital of Fudan University (EENT2015011), Shanghai, and complied with the Declaration of Helsinki. Informed consent was obtained from all the subjects. Patients with ARC were consecutively recruited from the Department of Ophthalmology &Visual Science, Eye & ENT Hospital of Fudan University between June 2016 and June 2017. Both newly diagnosed and referral ARC patients were included. Normal controls were consecutively recruited from individuals who participated in annual health screenings during the study period. The study clerks conducted face-to-face questionnaire interviews to gather data such as life habits, tobacco and alcohol intake, height, and body weight. The subjects were included according to inclusion criteria.

Medical examinations included assessments of electrocardiograms, X-rays, liver function, renal function, infectious disease, blood pressure, heart rate, and body temperature. All subjects underwent medical examinations at the Eye and ENT Hospital of Fudan University. Alcohol consumption (>3 times per week for more than six months), smoking (>1 cigarette per day and more than six months [current or former]) (self-reported), and dietary habits (meat [less vegetables]/vegetables [less meat]) were also collected. Furthermore, each ARC patient underwent a standardized ophthalmic examination performed by cataract specialists. A visual acuity measurement was obtained for each patient based on the International Standard Visual Acuity Chart. The details of the standardized ophthalmic examination have been previously described[13].

Inclusion criteria

ARC subjects

A total of 320 cataract subjects were recruited, of whom 101 (metabolic cataract = 15, traumatic cataract = 8, congenital cataract = 17, secondary cataract = 34, cancer = 3, renal disease = 3, liver disease = 5, other eye diseases = 16) were excluded from the

For peer review only - http://bmjopen.bmj.com/site/about/guidelines.xhtml

study based on the inclusion criteria. If both eyes suffered from ARC, only one eye was randomly selected for the study. The final sample consisted of 219 ARC patients who met the following inclusion criteria as described previously[13]: age ≥ 45 years; ARC recorded using the Lens Opacities Classification System III (LOCS III); ARC defined as the presence of any type of ARC (cortical, nuclear, or PSC) or a history of previous cataract surgery (pseudophakic or aphakic eyes) in either eye; no history of secondary cataracts, congenital cataracts, or any other eye diseases; and no history of systemic diseases such as acute infectious diseases, kidney disease, autoimmune disease, and cancer.

Control subjects

A total of 250 subjects were recruited. Of these, 32 normal subjects (cataract = 5, cancer = 2, renal disease = 6, liver disease = 4, autoimmune disease = 2, macular degeneration = 3, cardiovascular disease = 4, other diseases = 6) were later excluded from the study based on the inclusion criteria. The final sample consisted of 218 control subjects who met the following previously described criteria[13]: age ≥ 45 years; no history of any types of cataracts, secondary cataracts, congenital cataracts, and any other eye diseases; and no history of systemic diseases such as acute infectious diseases, kidney disease, autoimmune disease, and cancer.

Data collection

Clinical and demographic information were obtained from the medical data platform of Eye-ENT Hospital by trained staff (Shengjie Li and Mingxi Shao) using standardized data collection and quality control procedures, which produced reliable data for analysis.

For serum lipid measurements, blood samples were obtained in the morning after the subjects had fasted for 8 hours via standard venipuncture of the veins in the antecubital fossae. Blood coagulation was prevented using EDTA, and the obtained samples were centrifuged within 60 min (3000 rpm, 10 minutes, 20°C). Serum high-density lipoprotein cholesterol (HDL-C), low-density lipoprotein cholesterol (LDL-C), TG, and cholesterol (CHO) levels were measured by enzymatic colorimetry using a commercially available kit (Roche Diagnostics GmbH, Mannheim, Germany).

For peer review only - http://bmjopen.bmj.com/site/about/guidelines.xhtml

Serum apolipoprotein A (APOA) and B (APOB) levels were measured by immunoturbidimetry using a commercially available kit (Roche Diagnostics, GmbH, Mannheim, Germany). Internal controls were analyzed daily over a 2-year period, with typical monthly coefficient of variation of 2–5% and no significant changes in the values.

Statistical analyses

The results of this study are presented as mean ± standard deviation. Normality of the data was assessed with the Kolmogorov-Smirnoff test. Independent student's *t* test and chi-square test were used to compare the subject characteristics between the ARC and control groups. One-way analysis of variance was used to compare the serum lipid levels among the three groups (cortical, nuclear, and PSC). Based on the normal reference range value of LDL-C (LDL-C ≥ 3.36 mmol/L, LDL-C < 3.36 mmol/L), TG (TG ⩾ 2.26mmol/l, TG < 2.26 mmol/L), CHO (CHO ⩾ 5.20mmol/l, CHO<5.20mmol/l), and APOA (APOA ≥ 1.9 mmol/L, APOA < 1.9 mmol/L), the ARC and control subjects were divided into lower and higher groups, respectively. The prevalence of ARC in China is higher in females than in males (37.2% in men and 39.0% in women)[2]. Moreover, in our study, there were more female ARC subjects than male ARC subjects (125 vs. 94). Therefore, the subjects were categorized into gender subgroups. A two-sided $P < 0.05$ was considered statistically significant. Logistic regression analyses were performed to identify the association between serum lipid levels and ARC (control group = 0, ARC = 1; male = 0, female = 1). Odds ratios (ORs) with 95% confidence intervals (95%CI) were estimated by logistic regression analyses. All analyses were performed using SPSS 13.0 software (SPSS Inc., Chicago, IL, USA).

Results

A total of 219 ARC (male = 94, female = 125) subjects and 218 (male = 110, female = 118) normal control subjects were recruited in this study. There was no significant difference in the mean age, gender, smoking, and drinking between the ARC and control groups ($p > 0.005$). However, there was a significant difference in the body mass index (BMI), systolic blood pressure, diastolic blood pressure, and diet between

For peer review only - http://bmjopen.bmj.com/site/about/guidelines.xhtml

the ARC and control groups (p <0.05). Table 1 presents a summary of the demographics of the ARC and control groups. Supplementary figure 1 presents a summary of the corrletaions of individual subject's age with lipid profile.

Comparison of serum HDL-C, LDL-C, TG, CHO, APOA, and APOB levels between the ARC and control subjects

Table 2 shows the HDL-C, LDL-C, TG, CHO, APOA, and APOB levels in the ARC group. The level of serum LDL-C, TG, CHO, and APOA was significantly higher ($p < 0.05$) in the ARC group than in the control group.

Moreover, the ARC and control subjects were categorized into female and male subgroups. In both the female and male subgroups, the TG level was significantly higher ($p < 0.05$) in the ARC group than in the control group. In the male subgroup, the APOA level was significantly higher ($p < 0.05$) in the ARC group than in the control group. In the female subgroup, the level of LDL-C was significantly higher ($p < 0.05$) in the ARC group than in the control group.

Serum LDL-C, TG, CHO, and APOA levels in the ARC and control groups

The ARC and control subjects were each divided into a lower group and a higher group according to whether their serum LDL-C, TG, CHO, and APOA values were below or above the normal reference range, respectively (Table 3). Compared to the control group, more subjects from the ARC group were in the higher group (3.45, 17.37, 3.27, and11.91 fold, respectively, $p < 0.0001$).

Comparison of serum lipid levels in subjects with different types of ARC

Of the 219 ARC patients, 85 had nuclear cataracts, 108 had cortical cataracts, and 26 had PSC. Table 4 presents a comparison of the serum lipid levels and the demographic parameters in the ARC subjects. There were no statistically significant differences in the demographic and serum lipid levels among the nuclear, cortical, and PSC cataract groups ($p > 0.05$).

Logistic regression analysis of the association between serum lipid levels and ARC

Logistic regression analyses were performed to identify the association between serum HDL-C, LDL-C, TG, CHO, APOA, and APOB levels and ARC in comparison

For peer review only - http://bmjopen.bmj.com/site/about/guidelines.xhtml

to subjects without ARC (Table 5). Logistic regression analyses revealed that being female (OR = 1.719, 95% CI = 1.074–2.752) and BMI (OR = 1.297, 95% CI = 1.194–1.408), systolic blood pressure (OR = 1.029, 95% CI = 1.008–1.051), LDL-C (OR = 1.897, 95% CI = 0.960–3.678), and TG levels (OR = 1.854, 95% CI = 1.232–2.791) were associated with ARC after adjusting for age and other demographic parameters.

Discussion

In this case-control study of the Chinese Han population, we found the following: First, the serum LDL-C, TG, CHO, and APOA levels was significantly higher ($p < 0.05$) in the ARC group than in the control group. Second, the proportion of individuals with higher LDL-C, TG, CHO, and APOA levels was higher in the ARC group (3.45, 17.37, 3.27, and 11.91-fold, $p < 0.0001$) relative to the control group. Finally, high serum LDL-C (OR = 1.897, 95% CI = 0.960–3.678), and TG levels (OR = 1.854, 95% CI = 1.232–2.791) were associated with ARC after adjusting for age, gender, and other demographic parameters. The results of this case-control study indicate that higher serum LDL-C and TG levels are associated with a significantly increased risk of ARC.

In a study on the association of metabolic syndrome and ARC, reduced HDL-C and elevated TG levels were found to be significantly associated with cataract in women[14]. In a population-based study (n = 4,926) in Beaver Dam, Klein et al.[15] reported that higher serum HDL-C levels were associated with decreased risk of cataract. Moreover, an extremely strong association ($p < 0.0001$) between HDL-C levels and the development of lens opacities was also reported in the South African population[16]. Furthermore, an elevated level of serum TG was significantly related to cataract in some studies[7,17]. In the present study, we also found that serum TG concentration was higher in the ARC group than in the normal group and that TG concentration was a risk factor of ARC, but the HDL-C level did not significantly differ between groups. Sabanayagam et al.[4] also found no significant associations between serum HDL-C and cataract. The mechanism linking hyperlipidemia and cataract is not clear. Thus far, no studies have clarified how changes in blood lipid affect the onset and/or development

For peer review only - http://bmjopen.bmj.com/site/about/guidelines.xhtml

of ARC. Animal studies have shown that hyperlipidemia and low HDL-C are associated with an earlier onset and an elevated incidence of diabetic cataracts[18], and inflammation and oxidative stress resulting from low HDL-C levels could induce cataract formation[19]. Moreover, it has been suggested that hyperlipidemia is related to ARC through its associated complications such as cardiovascular disease[9,15].

In the female and male ARC subgroups, the TG level was significantly higher ($p < 0.05$) in the ARC group than in the control group. However, the APOA level was significantly higher in the ARC group than in the control group in the male subgroup but not in the female subgroup. In the female subgroup, the LDL-C level was significantly higher in the ARC group than in the control group but not in the male subgroup. Tavani et al.[20] reported that hyperlipidemia (OR, 1.8; 95% CI, 1.2–2.7) was associated with an increased risk of cataract in women from northern Italy. In addition, elevated TG was also significantly associated with cataract in women but not in men[14]. A consistent gender difference in the association between blood lipid concentration and cataract was observed. Differences of hormonal[21] and life-style related characteristics[22] between men and women may explain this gender difference.

In the subgroup analysis for ARC subtype, no statistically significant difference in the demographic and serum lipid levels was found among the nuclear, cortical, and PSC cataract groups ($p > 0.05$). Hiller et al.[7] found that HDL-C levels were associated with PSC cataract but not in cortical and nuclear cataract. Moreover, Park et al.[14] reported that reduced HDL-C was only significantly associated with nuclear cataract. The results of these existing studies are controversial, and the potential pathogenesis was unclear.

Our study also found that female gender (OR = 1.719, 95% CI = 1.074–2.752), high BMI (OR = 1.297, 95% CI = 1.194–1.408), and high systolic blood pressure (OR = 1.029, 95% CI = 1.008–1.051), which were closely associated with serum lipid concentration, were associated with an increased risk of ARC after adjusting for age and other demographic parameters. In the Blue Mountains Eye Study, obesity (BMI ≥30 kg/m^2) and hypertension were significantly associated with an increased incidence of cataract[23]. Moreover, Jacques et al.[6] also reported that women with a

For peer review only - http://bmjopen.bmj.com/site/about/guidelines.xhtml

BMI of ≥30 had a higher prevalence of PSC opacities than women with a BMI of <25 (OR: 2.5).

We acknowledge that our study has some limitations. First, as this is a cross-sectional, case-control study, our ability to explore the exact mechanisms underlying the associations between serum lipid concentration and ARC remained limited. This in turn limited our ability to make exact causal inferences between serum lipid concentration and ARC. Second, the subjects involved in the study are from a racially homogeneous background (all Chinese), which may limit the generalizability of the results. Third, we did not consider treatment with lipid-lowering medications and how it might affect the results. Last, the methods used to analyze lipid levels in the blood are superficial. a more sophisticated GC-MS approach should be used in the future study. Therefore, a multicenter study with a larger sample size should be conducted in the future.

In conclusion, the findings from this case-control study suggest that the serum LDL-C, TG, CHO, and APOA concentrations were significantly higher in the ARC patients than in the controls. The serum LDL-C and TG concentration were significantly associated with ARC. In addition, no significant difference in the serum lipid levels was found between the nuclear, cortical, and PSC cataract groups. These findings indicate a need for health promotional activities aimed at controlling blood lipid concentration among high-risk populations.

References

1. Bourne RRA, Stevens GA, White RA, et al. Causes of vision loss worldwide, 1990-2010: a systematic analysis. *Lancet Glob Health*. 2013;1(6):e339-349. doi:10.1016/S2214-109X(13)70113-X.

2. Tang Y, Wang X, Wang J, et al. Prevalence of Age-Related Cataract and Cataract Surgery in a Chinese Adult Population: The Taizhou Eye Study. *Invest Ophthalmol Vis Sci*. 2016;57(3):1193-1200. doi:10.1167/iovs.15-18380.

3. Klein BE, Klein R, Linton KL. Prevalence of age-related lens opacities in a population. The Beaver Dam Eye Study. *Ophthalmology*. 1992;99(4):546-552.

4. Sabanayagam C, Wang JJ, Mitchell P, et al. Metabolic syndrome components and age-related cataract: the Singapore Malay eye study. *Invest Ophthalmol Vis*

For peer review only - http://bmjopen.bmj.com/site/about/guidelines.xhtml

Sci. 2011;52(5):2397-2404. doi:10.1167/iovs.10-6373.

5. Hennis A, Wu S-Y, Nemesure B, Leske MC, Barbados Eye Studies Group. Risk factors for incident cortical and posterior subcapsular lens opacities in the Barbados Eye Studies. *Arch Ophthalmol Chic Ill 1960*. 2004;122(4):525-530. doi:10.1001/archopht.122.4.525.

6. Jacques PF, Moeller SM, Hankinson SE, et al. Weight status, abdominal adiposity, diabetes, and early age-related lens opacities. *Am J Clin Nutr*. 2003;78(3):400-405.

7. Hiller R, Sperduto RD, Reed GF, D'Agostino RB, Wilson PWF. Serum lipids and age-related lens opacities: a longitudinal investigation: the Framingham Studies. *Ophthalmology*. 2003;110(3):578-583. doi:10.1016/S0161-6420(02)01762-1.

8. Marks RG, Hale WE, Perkins LL, May FE, Stewart RB. Cataracts in Dunedin Program participants: an evaluation of risk factors. *J Cataract Refract Surg*. 1988;14(1):58-63.

9. Jahn CE, Janke M, Winowski H, von Bergmann K, Leiss O, Hockwin O. Identification of metabolic risk factors for posterior subcapsular cataract. *Ophthalmic Res*. 1986;18(2):112-116.

10. Heydari B, Kazemi T, Zarban A, Ghahramani S. Correlation of cataract with serum lipids, glucose and antioxidant activities: a case-control study. *West Indian Med J*. 2012;61(3):230-234.

11. Clayton RM, Cuthbert J, Duffy J, et al. Some risk factors associated with cataract in S.E. Scotland: a pilot study. *Trans Ophthalmol Soc U K*. 1982;102 Pt 3:331-336.

12. Klein BE, Klein R, Lee KE. Diabetes, cardiovascular disease, selected cardiovascular disease risk factors, and the 5-year incidence of age-related cataract and progression of lens opacities: the Beaver Dam Eye Study. *Am J Ophthalmol*. 1998;126(6):782-790.

13. Shao M, Li D, Teng J, Zhang Y, Li S, Cao W. Association Between Serum Complement C3 Levels and Age-Related Cataract. *Invest Ophthalmol Vis Sci*. 2017;58(11):4934-4939. doi:10.1167/iovs.17-22522.

14. Park Y-H, Shin JA, Han K, Yim HW, Lee W-C, Park Y-M. Gender difference in the association of metabolic syndrome and its components with age-related cataract: the Korea National Health and Nutrition Examination Survey 2008-2010. *PloS One*. 2014;9(1):e85068. doi:10.1371/journal.pone.0085068.

15. Klein BE, Klein R, Lee KE. Cardiovascular disease, selected cardiovascular

For peer review only - http://bmjopen.bmj.com/site/about/guidelines.xhtml

disease risk factors, and age-related cataracts: the Beaver Dam Eye Study. *Am J Ophthalmol*. 1997;123(3):338-346.

16. Meyer D, Parkin D, Maritz FJ, Liebenberg PH. Abnormal serum lipoprotein levels as a risk factor for the development of human lenticular opacities. *Cardiovasc J South Afr Off J South Afr Card Soc South Afr Soc Card Pract*. 2003;14(2):60-64.

17. Paunksnis A, Bojarskiene F, Cimbalas A, Cerniauskiene LR, Luksiene DI, Tamosiunas A. Relation between cataract and metabolic syndrome and its components. *Eur J Ophthalmol*. 2007;17(4):605-614.

18. Tsutsumi K, Inoue Y, Yoshida C. Acceleration of development of diabetic cataract by hyperlipidemia and low high-density lipoprotein in rats. *Biol Pharm Bull*. 1999;22(1):37-41.

19. von Eckardstein A, Hersberger M, Rohrer L. Current understanding of the metabolism and biological actions of HDL. *Curr Opin Clin Nutr Metab Care*. 2005;8(2):147-152.

20. Tavani A, Negri E, La Vecchia C. Selected diseases and risk of cataract in women. A case-control study from northern Italy. *Ann Epidemiol*. 1995;5(3):234-238.

21. Baños G, Guarner V, Pérez-Torres I. Sex steroid hormones, cardiovascular diseases and the metabolic syndrome. *Cardiovasc Hematol Agents Med Chem*. 2011;9(3):137-146.

22. Lee DH, Kim YM, Jekal Y, et al. Low levels of physical activity are associated with increased metabolic syndrome risk factors in korean adults. *Diabetes Metab J*. 2013;37(2):132-139. doi:10.4093/dmj.2013.37.2.132.

23. al YC et. Cardiovascular disease, vascular risk factors and the incidence of cataract and cataract surgery: the Blue Mountains Eye Study. - PubMed - NCBI. https://www.ncbi.nlm.nih.gov/pubmed/14628965. Accessed November 18, 2017.

For peer review only

For peer review only - http://bmjopen.bmj.com/site/about/guidelines.xhtml

Figure Legends

Supplementary Figure 1. The correlations of individual ARC's age with lipid profile.

For peer review only

For peer review only - http://bmjopen.bmj.com/site/about/guidelines.xhtml

Tables

Table 1. Demographics of subjects between ARC and control group

	ARC group (n=219)	Control group (n=218)	t value	P value
Age (year)	66.99±8.83 (46-85)	66.00±7.10 (48-79)	1.281	0.201
Gender (Male/Female)	94/125	110/118	1.276	0.259
BMI (kg/m^2)	23.68±3.16	21.55±2.74	7.476	<0.001
SBP (mmHg)	132.45±15.50	129.92±5.55	2.230	0.027
SDP (mmHg)	74.21±10.65	76.58±5.37	2.896	0.004
IOP (mmHg)	13.84±3.59	-	-	-
Visual acuity	0.23±0.15	-	-	-
Smoking (yes/no)	15/204	18/200	0.310	0.578
Drinking (yes/no)	33/186	23/195	1.996	0.158
Diet (meat/vegetarian)	159/60	98/120	34.476	<0.001

ARC: age-related cataract, BMI: body mass index, SBP: systolic blood pressure, SDP: diastolic blood pressure, IOP: intraocular pressure. Data are expressed as mean±standard deviation (SD). Independent-Samples T Test and chi-square test was used.

Table 2. Comparison of serum lipid levels between ARC and control group, stratified according to gender

	ARC group	Control group	t value	P value
LDL-C (mmol/l)	2.65±0.93	2.46±0.64	2.461	0.014
Male	2.45±0.85	2.43±0.60	0.184	0.854
Female	2.82±0.97	2.49±0.67	2.894	0.004
TG (mmol/l)	1.56±1.25	1.18±0.35	4.238	<0.001
Male	1.54±1.42	1.15±0.36	2.609	0.010
Female	1.57±1.10	1.22±0.35	3.364	0.001
HDL-C (mmol/l)	1.14±0.43	1.19±0.28	1.201	0.231
Male	1.10±0.48	1.10±0.28	0.064	0.949
Female	1.18±0.39	1.28±0.25	2.158	0.032
APOA (mmol/l)	1.34±0.23	1.28±0.36	2.270	0.024
Male	1.30±0.23	1.22±0.36	1.983	0.048
Female	1.39±0.22	1.33±0.35	1.649	0.101
APOB (mmol/l)	0.85±0.26	0.86±0.19	0.772	0.441
Male	0.81±0.25	0.83±0.18	0.757	0.450
Female	0.87±0.26	0.89±0.19	0.590	0.556
CHO (mmol/l)	4.47±1.78	4.26±0.79	2.146	0.032
Male	4.21±1.10	4.03±0.69	1.438	0.152
Female	4.67±1.20	4.49±0.81	1.275	0.204

ARC: age-related cataract, LDL-C: low density lipoprotein cholesterol, HDL-C: high density lipoprotein cholesterol, TG: triglyceride, APOA: apoA apolipoprotein, APOB: apoB apolipoprotein, CHO: cholesterol. Data are expressed as mean±standard

For peer review only - http://bmjopen.bmj.com/site/about/guidelines.xhtml

deviation (SD). Independent-Samples T Test was used.

Table 3. The number of subjects in ARC and control group, according to LDL-C, TG, CHO, and APOA levels, respectively

	ARC group	Control group	Fold (t value)	P value
LDL-C (mmol/l)				
<3.36	167	203		
⩾3.36	52 (52/219=23.74%)	15 (15/218=6.88%)	3.45 (17.818)	<0.001
TG (mmol/l)				
<2.26	184	216		
⩾2.26	35 (35/219=15.98%)	2 (2/218=0.92%)	17.37 (27.136)	<0.001
CHO (mmol/l)				
<5.20	153	198		
⩾5.20	66 (66/219=30.14%)	20 (20/218=9.17%)	3.27 (20.549)	<0.001
APOA (mmol/l)				
<1.9	207	217		
⩾1.9	12 (12/219=5.48%)	1 (1/218=0.46%)	11.91 (8.996)	<0.001

ARC: age-related cataract, LDL-C: low density lipoprotein cholesterol, TG: triglyceride, APOA: apoA apolipoprotein, CHO: cholesterol. Chi-square test was used.

Table 4. Comparison of serum lipid levels in subjects with different types of ARC

	Nuclear cataract	Cortical cataract	PSC	t value	P value
Age (year)	67.54±8.83	66.59±8.55	66.87±10.39	0.282	0.754
Gender (Male/Female)	33/52	49/59	12/14	0.985	0.619
BMI (kg/m^2)	23.62±3.24	23.65±3.14	24.13±3.04	0.219	0.804
SBP (mmHg)	134.28±16.65	131.15±14.38	131.63±16.61	1.006	0.367
SDP (mmHg)	74.22±8.28	73.65±10.67	77.94±18.93	1.132	0.324
LDL-C (mmol/l)	2.67±0.97	2.60±0.87	2.81±1.08	0.491	0.612
TG (mmol/l)	1.46±1.28	1.68±1.32	1.38±0.64	1.022	0.362
HDL-C (mmol/l)	1.18±0.39	1.11±0.45	1.18±0.46	0.784	0.458
APOA (mmol/l)	1.29±0.34	1.27±0.38	1.29±0.35	0.107	0.899
APOB (mmol/l)	0.85±0.26	0.83±0.25	0.87±0.31	0.341	0.712
CHO (mmol/l)	4.45±1.22	4.47±1.11	4.51±1.38	0.020	0.981

ARC: age-related cataract, PSC: posterior subcapsular, BMI: body mass index, SBP: systolic blood pressure, SDP: diastolic blood pressure, LDL-C: low density lipoprotein cholesterol, HDL-C: high density lipoprotein cholesterol, TG: triglyceride, APOA: apoA apolipoprotein, APOB: apoB apolipoprotein, CHO: cholesterol. Data

For peer review only - http://bmjopen.bmj.com/site/about/guidelines.xhtml

For peer review only

are expressed as mean±standard deviation (SD). Independent-Samples T Test and chi-square test was used.

Table 5. Logistic regression analysis of the association between serum lipid levels and ARC

	B	OR	P value	95%CI
Female	0.542	1.719	0.024	1.074-2.752
BMI (kg/m^2)	0.260	1.297	<0.001	1.194-1.408
SBP (mmHg)	0.029	1.029	0.006	1.008-1.051
LDL-C (mmol/l)	0.631	1.897	0.046	0.960-3.678
TG (mmol/l)	0.617	1.854	0.003	1.232-2.791

ARC: age-related cataract, BMI: body mass index, SBP: systolic blood pressure, LDL-C: low density lipoprotein cholesterol, TG: triglyceride, 95%CI: 95%confidence interval. Logistic regression analyses were performed to identify the association between serum lipid levels and ARC (control group=0, ARC=1; male=0, female=1) after adjusting for age, smoking, drinking, diastolic blood pressure, diet, high density lipoprotein cholesterol, apoA apolipoprotein, apoB apolipoprotein, and cholesterol.

For peer review only

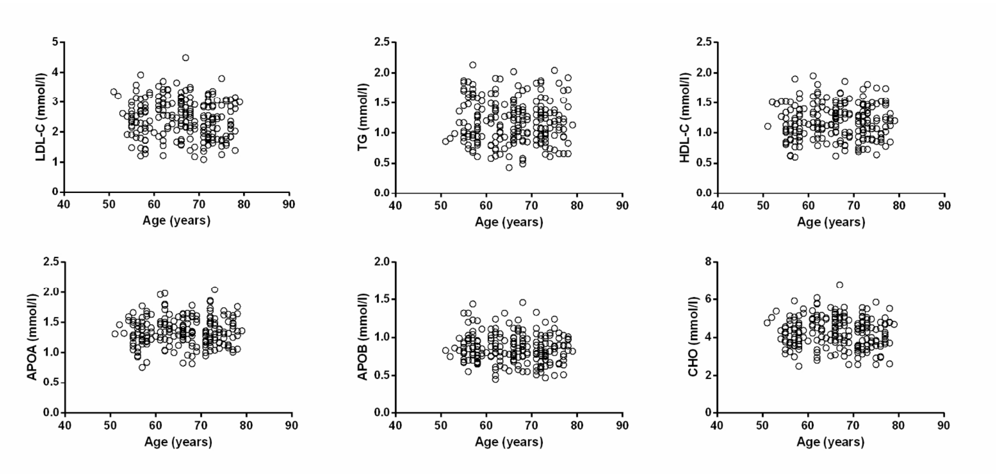

199x94mm (300 x 300 DPI)

For peer review only

STROBE 2007 (v4) Statement—Checklist of items that should be included in reports of *case-control studies*

Section/Topic	Item #	Recommendation	Reported on page #
**Title and abstract**	1	Association between serum lipids concentration and patients with age-related cataract in China: a cross-sectional, case-control study	1
		Objective: Obesity and a high-fat diet have been found to be associated with an increased risk of age-related cataract (ARC). Thus, this study aimed to investigate whether serum lipid levels are associated with the incidence of ARC.<br>Design: Cross-sectional, case-control study.<br>Setting: Eye & ENT Hospital of Fudan University, Shanghai, China.<br>Participants: A total of 219 ARC (male = 94, female = 125) subjects and 218 (male = 110, female = 118) normal control subjects were recruited in this study.<br>Outcome measures: A detailed eye and systematic examination was performed. Serum high-density lipoprotein cholesterol (HDL-C), low-density lipoprotein cholesterol (LDL-C), triglyceride (TG), and cholesterol (CHO) levels were measured by enzymatic colorimetry, and serum apolipoprotein A (APOA) and apoB (APOB) levels were measured by immunoturbidimetry. The subgroups were classified according to gender and types of disease (cortical, nuclear, and posterior subcapsular cataract). Logistic regression analyses were performed to identify the association between serum lipid levels and ARC.<br>Results: The serum LDL-C, TG, CHO, and APOA levels were significantly higher ($p < 0.05$) in the ARC group than in the control group. A similar result was observed when the serum lipid concentrations were compared between the ARC and control groups both in male and female subgroups. A higher proportion of individuals in the ARC group had higher LDL-C, TG, CHO, and APOA levels (fold = 3.45, 17.37, 3.27, and11.91, respectively; $p < 0.0001$ in all cases) than in the control group. Results of the logistic regression analyses revealed that high LDL-C (Odds ratios [ORs] = 1.897, 95% CI = 0.960–3.678), and TG (OR = 1.854, 95% CI = 1.232–2.791) were the independent risk factors for ARC.<br>Conclusion: The serum LDL-C and TG levels were demonstrated to be independent risk factors for ARC.	2
**Introduction**			
Background/rationale	2	ARC is a complex diseases with multiple risk components, including high myopia, diabetes, hyperglycemia, obesity, blood pressure, lipids. Among these risk factors, obesity, blood pressure, and lipids have been found to be consistently associated with blood lipid concentration.	3
Objectives	3	Here, we conducted a large, cross-sectional, case-control study to detect and compare the blood lipid levels between ARC patients and normal subjects and to investigate the possible association between blood lipid levels and the risk of ARC.	3-4

For peer review only

**Methods**			
Study design	4	Cross-sectional, case-control study	4
Setting	5	Patients with ARC were consecutively recruited from the Department of Ophthalmology &Visual Science, Eye & ENT Hospital of Fudan University between June 2016 and June 2017.	4
Participants	6	*Both newly diagnosed and referral ARC patients were included.*	4
		Normal controls were consecutively recruited from individuals who participated in annual health screenings during the study period.	4
Variables	7	Medical examinations included assessments of electrocardiograms, X-rays, liver function, renal function, infectious disease, blood pressure, heart rate, and body temperature. Serum high-density lipoprotein cholesterol (HDL-C), low-density lipoprotein cholesterol (LDL-C), TG, and cholesterol (CHO) levels were measured by enzymatic colorimetry using a commercially available kit (Roche Diagnostics GmbH, Mannheim, Germany). Serum apolipoprotein A (APOA) and B (APOB) levels were measured by immunoturbidimetry using a commercially available kit (Roche Diagnostics, GmbH, Mannheim, Germany).	5-6
Data sources/ measurement	8*	Serum high-density lipoprotein cholesterol (HDL-C), low-density lipoprotein cholesterol (LDL-C), TG, and cholesterol (CHO) levels were measured by enzymatic colorimetry using a commercially available kit (Roche Diagnostics GmbH, Mannheim, Germany). Serum apolipoprotein A (APOA) and B (APOB) levels were measured by immunoturbidimetry using a commercially available kit (Roche Diagnostics, GmbH, Mannheim, Germany). Internal controls were analyzed daily over a 2-year period, with typical monthly coefficient of variation of 2–5% and no significant changes in the values.	5-6
Bias	9	Inclusion Criteria	4-5
Study size	10	A total of 320 cataract subjects were recruited, of whom 101 (metabolic cataract = 15, traumatic cataract = 8, congenital cataract = 17, secondary cataract = 34, cancer = 3, renal disease = 3, liver disease = 5, other eye diseases = 16) were excluded from the study based on the inclusion criteria.	4-5
Quantitative variables	11	Based on the normal reference range value of LDL-C (LDL-C ⩾ 3.36 mmol/L, LDL-C < 3.36 mmol/L), TG (TG⩾2.26mmol/l, TG < 2.26 mmol/L), CHO (CHO⩾5.20mmol/l, CHO<5.20mmol/l), and APOA (APOA ⩾ 1.9 mmol/L, APOA < 1.9 mmol/L), the ARC and control subjects were divided into lower and higher groups, respectively. The prevalence of ARC in China is higher in females than in males (37.2% in men and 39.0% in women)2. Moreover, in our study, there were more female ARC subjects than male ARC subjects (125 vs. 94). Therefore, the subjects were categorized into gender subgroups.	6
Statistical methods	12	(*a*) the Kolmogorov-Smirnoff test,	6
		(*b*) The independent student's t-test and chi-square test,	6
		(*c*) The one-way ANOVA test,	6
		(*d*) Multivariate logistic regression analyses	6
		(*e*) None	

For peer review only

**Results**			
Participants	13*	(a) A total of 219 ARC (male = 94, female = 125) subjects and 218 (male = 110, female = 118) normal control subjects were recruited in this study.	6
		(b) none	
		(c) none	
Descriptive data	14*	(a) table1	table1
		(b)none	
Outcome data	15*	none	
Main results	16	(*a*) The serum LDL-C, TG, CHO, and APOA levels were significantly higher ($p < 0.05$) in the ARC group than in the control group. A similar result was observed when the serum lipid concentrations were compared between the ARC and control groups both in male and female subgroups. A higher proportion of individuals in the ARC group had higher LDL-C, TG, CHO, and APOA levels (fold = 3.45, 17.37, 3.27, and11.91, respectively; $p < 0.0001$ in all cases) than in the control group. Results of the logistic regression analyses revealed that high LDL-C (Odds ratios [ORs] = 1.897, 95% CI = 0.960 ‐ 3.678), and TG (OR = 1.854, 95% CI = 1.232 ‐ 2.791) were the independent risk factors for ARC.	6-8
		(*b*) Comparison of laboratory findings in ARC and control group	6-8
		(*c*) none	
Other analyses	17	Comparison of serum lipid levels in subjects with different types of ARC	7
**Discussion**			
Key results	18	Results of the logistic regression analyses revealed that high LDL-C (Odds ratios [ORs] = 1.897, 95% CI = 0.960–3.678), and TG (OR = 1.854, 95% CI = 1.232–2.791) were the independent risk factors for ARC.	8
Limitations	19	First, as this is a cross-sectional, case-control study, our ability to explore the exact mechanisms underlying the associations between serum lipid concentration and ARC remained limited. This in turn limited our ability to make exact causal inferences between serum lipid concentration and ARC. Second, the subjects involved in the study are from a racially homogeneous background (all Chinese), which may limit the generalizability of the results. Third, we did not consider treatment with lipid-lowering medications and how it might affect the results. Last, the methods used to analyze lipid levels in the blood are superficial. a more sophisticated GC-MS approach should be used in the future study.	10
Interpretation	20	Give a cautious overall interpretation of results considering objectives, limitations, multiplicity of analyses, results from similar studies, and other relevant evidence	8-10
Generalisability	21	Discuss the generalisability (external validity) of the study results	8-10
**Other information**			
Funding	22	This research project was supported by the Shanghai Municipal Commission of Health and Family Planning (20174Y0169)	1

For peer review only

*Give information separately for cases and controls in case-control studies and, if applicable, for exposed and unexposed groups in cohort and cross-sectional studies.

Note: An Explanation and Elaboration article discusses each checklist item and gives methodological background and published examples of transparent reporting. The STROBE checklist is best used in conjunction with this article (freely available on the Web sites of PLoS Medicine at http://www.plosmedicine.org/, Annals of Internal Medicine at http://www.annals.org/, and Epidemiology at http://www.epidem.com/). Information on the STROBE Initiative is available at www.strobe-statement.org.

For peer review only
